# Analysis of prosthetic outcomes and influencing factors in patients with alveolar bone atrophy

**DOI:** 10.2340/aos.v85.46467

**Published:** 2026-07-20

**Authors:** Wei Zhang, Chang’ao Xue, Yan Chen

**Affiliations:** Department of Stomatology, Nanjing First Hospital, Nanjing Medical University, Nanjing, China

**Keywords:** Alveolar bone atrophy, dental implantation, implant-supported prosthesis, influencing factors, retrospective study

## Abstract

**Objective:**

To investigate implant-supported prosthetic outcomes and influencing factors in alveolar bone atrophy.

**Material and methods:**

One hundred and fifty patients with atrophy receiving implants (2021–2022) were followed for 2 years. Success (*n* = 97, 64.7%) and failure (*n* = 53, 35.3%) groups were compared using univariate analysis, followed by multivariate logistic regression to identify factors associated with overall failure and Cox regression to assess time to failure.

**Results:**

Univariate analysis identified age, gender, smoking, implant length, and prosthesis type as significant (*p* < 0.05). Multivariate logistic regression showed short implant length (< 10 mm) as the sole independent risk factor for failure (OR = 4.418, *p* = 0.004); age ≥ 60 years (OR = 2.560, *p* = 0.094) and female gender (OR = 1.127, *p* = 0.807) were not significant. Multivariable Cox regression revealed age ≥ 60 years as a significant predictor of earlier failure (HR = 2.558, *p* = 0.010), while female gender and short implants were not (*p* > 0.05). Kaplan–Meier curves showed significant survival differences for age (*p* = 0.010), gender (*p* = 0.023), and implant length (*p* = 0.021).

**Conclusion:**

Short implant independently increases failure risk; age ≥ 60 years accelerates failure. Bone augmentation is preferred. Findings need validation.

## Introduction

With the rapid advancement of dental implant technology, implant-supported prostheses have emerged as the preferred treatment option for partial and complete edentulism due to their superior esthetics, comfort, and masticatory efficiency. Research indicates that post-extraction alveolar bone resorption is cumulative, progressive, and irreversible. The incidence of alveolar bone atrophy increases over time following implant restoration, reaching 21% at 3 months, 36% at 6 months, and as high as 44% by 12 months [1]. Notably, severe alveolar bone atrophy is more prevalent in elderly patients with chronic periodontitis or long-term edentulism, and the combined effects of maxillary sinus pneumatization further reduce available bone height, with up to 30–40% of edentulous maxilla patients facing insufficient bone volume challenges [2]. Moreover, clinical observations confirm that alveolar bone resorption accelerates in the first 3 months post-extraction and gradually slows after 6 months, but the irreversible nature of this process still significantly limits subsequent implant treatment options [3].

While conventional implant techniques have achieved favorable outcomes in patients with sufficient bone volume, cases involving alveolar bone atrophy continue to present significant challenges, including surgical complexity, prolonged osseointegration periods, and suboptimal long-term stability [4]. Although bone augmentation therapies including guided bone regeneration (GBR), maxillary sinus floor elevation, and block bone grafting offer viable solutions for patients with inadequate bone volume, these procedures are technically demanding, extend the healing period, increase treatment costs, and thus fall short of fully addressing clinical needs [5]. A clinical study demonstrated that GBR combined with transalveolar sinus floor elevation (TSFE) can achieve significant vertical bone augmentation (up to 9.67 mm increase in bone height), but this complex combined approach still requires strict patient selection and long-term follow-up to maintain bone volume stability [6]. Current research has largely explored the effect of bone augmentation on implant outcomes among patients with existing alveolar atrophy, but the applicability of these techniques in high-risk populations remains understudied.

However, the failure rate of implant-supported prostheses in patients with alveolar bone atrophy remains as high as 15–30%, especially in elderly, smoking populations, and patients with uncontrolled systemic diseases [5]. Recent evidence confirms that smoking, poorly controlled diabetes, and immune system disorders significantly inhibit oral blood circulation and tissue repair, further increasing implant failure risk in patients with bone atrophy [6]. Although existing studies have explored the impact of individual factors on implant outcomes, the synergistic effects of multiple risk factors (e.g. the combined effect of advanced age, female gender, and poor bone quality), time-dependent effects, and their association with bone atrophy grades in patients with alveolar bone atrophy have not been clearly elucidated [5]. This knowledge gap prevents precise clinical risk prediction and personalized treatment, leading to suboptimal therapeutic effects in high-risk overlapping populations.

Therefore, the innovativeness of this study lies in its retrospective analysis of clinical data from 150 patients with alveolar bone atrophy over a 2-year period. By comprehensively evaluating the effects of systemic factors, local bone conditions, surgical protocols, and prosthetic design on implant outcomes, this study for the first time focuses on the ‘alveolar bone atrophy + advanced age/female’ high-risk overlapping population and reveals the protective effect of short implants, modifying the previous understanding of short implants in atrophic bone cases, and proposes a ‘two-risk + one-protective’ early warning and intervention model, aiming to provide a more targeted scientific basis for individualized treatment strategies in this patient population.

## Materials and methods

### Study design and setting

This retrospective cohort study followed the STROBE (Strengthening the Reporting of Observational Studies in Epidemiology) reporting guideline. A consecutive sampling method was employed to select patients with alveolar bone atrophy who underwent implant placement and subsequent prosthetic restoration in the Department of Stomatology at Nanjing First Hospital, Nanjing Medical University during January 2021–December 2022. The inclusion criteria were: (1) an age ≥ 18 years; (2) receiving first-time implant-supported restoration for single tooth gaps or partial edentulism; (3) a preoperative cone beam computed tomography (CBCT) confirmation of alveolar bone atrophy, classified according to the Lekholm & Zarb bone quality classification (types B, C, and D) and the Terheyden bone atrophy classification (grades 1–4) [7]; (4) completion of the prosthetic phase following implant surgery, with availability of complete and standardized clinical and radiographic data; and (5) provision of informed consent. Exclusion criteria comprised: (1) severe hematological disorders, malignant tumors, history of head and neck radiotherapy, uncontrolled severe cardiocerebrovascular diseases, or psychiatric conditions; (2) untreated severe periodontitis or presence of active oral infection; (3) use of anti-inflammatory medications within the preceding 30 days; (4) women who were lactating, pregnant, menstruating, or patients on bisphosphonates; and (5) presence of malocclusion or limited mouth opening.

Sample size was determined following Kendall’s principle (5–10 times the number of variables). With 15 variables, the upper limit (150 patients) was targeted. PASS 15.0 software confirmed that 150 cases exceeded the minimum required sample size of 128 cases (α = 0.05, β = 0.1, OR = 2.0) [8].

### Outcome definition

At the 2-year follow-up, prosthetic outcomes were classified according to the composite criteria proposed by Papaspyridakos et al. [9], encompassing four domains: (1) implant-level (no mobility, no peri-implant radiolucency, marginal bone loss < 2 mm after first year of loading); (2) peri-implant soft tissue (no bleeding on probing, probing depth ≤ 5 mm); (3) prosthetic-level (no screw loosening, no fracture of prosthesis, abutment, or veneer); (4) patient-reported satisfaction (function and esthetics). This was assessed using a visual analog scale (VAS, 0–10) at the 2-year follow-up, where patients rated their satisfaction with masticatory function (0 = completely dissatisfied, 10 = completely satisfied) and esthetics (0 = completely dissatisfied, 10 = completely satisfied). A score ≥ 8 in both domains was considered satisfactory. Prosthetic success required fulfillment of all four domains. Failure was defined as non-fulfillment of any domain, including implant loss. Specifically, if a patient failed to meet any one of the four composite criteria (implant-level, peri-implant soft tissue, prosthetic-level, or patient-reported satisfaction), the case was classified as failure. For example, even if implant osseointegration was successful but the patient reported low satisfaction (VAS < 8 for function or esthetics), the case was still categorized as failure.

Level of analysis: This study was conducted at the patient level, as each patient contributed one implant-supported prosthesis. All outcome assessments (implant-level, peri-implant soft tissue, prosthetic-level, and patient-reported satisfaction) were integrated into a composite outcome at the patient level. Failure was defined as non-fulfillment of any domain for that patient.

### Data collection and variables

The following information was collected from the electronic medical record system and radiographic archives:

1. Patient demographics and history: Age, gender, smoking status (current smoker/former smoker/never smoker), and presence of diabetes mellitus (yes/no).
2. Local bone conditions: Preoperative CBCT scans were used to assess bone quantity according to the Terheyden classification (grades 1–4) [10] and bone quality according to the Lekholm & Zarb classification (types I–IV: I = pure cortical, II = mostly cortical, III = mostly cancellous, IV = loose cancellous bone) [11].
3. Surgical factors: Surgical approach (GBR/maxillary sinus floor elevation/block bone graft), implant insertion direction (vertical/tilted, determined on postoperative panoramic radiographs or CBCT: vertical if the implant axis was within 15° of the occlusal plane perpendicular, tilted if >15°), implant length (short implant < 10 mm/long implant ≥ 10 mm), implant diameter (< 3.6 mm/3.6–4.2 mm/> 4.2 mm), implant surface technology, surgical protocol (immediate loading or not), and presence of intraoperative or postoperative complications (yes/no) [12, 13].
4. Prosthetic factors: Type of prosthesis (single crown/fixed partial denture (FPD) with splinted crowns/FPD), cuspal inclination (25°/35°/45°, measured using a goniometer on the articulated diagnostic cast or intraoral digital scan), and occlusal scheme (group function/canine guidance) [13].
5. Oral hygiene status: The modified plaque index (MPI) was used to evaluate plaque accumulation around the implants, scored on a 0–3 scale: 0 = no plaque detected; 1 = plaque not visible by the naked eye but detectable with a probe; 2 = moderate accumulation of visible plaque; 3 = abundant soft deposits [14].
6. Bone augmentation adequacy: According to the ITI consensus recommendation, all implant placements required a minimum of 1.5 mm of native or augmented bone surrounding the entire implant shoulder. In patients with insufficient residual bone volume, bone augmentation procedures (GBR, maxillary sinus floor elevation, or block bone graft) were performed. Patients in whom adequate bone augmentation could not be achieved were excluded from the study.

### Ethical approval

The study protocol obtained approval from the Ethics Committee of Nanjing First Hospital, Nanjing Medical University (No. KY20250901-KS-03; Ethics vote date: March 15, 2024) and was conducted in accordance with the Declaration of Helsinki. Due to the study’s retrospective design, the ethics committee granted a waiver for obtaining written informed consent. All patient medical records were anonymized to protect privacy.

### Statistical analysis

Data analysis was performed using SPSS software (version 26.0). Categorical data are presented as frequency counts and percentages (*n* [%]), and intergroup comparisons were conducted using the chi-square (χ²) test.

Variable assignments: For statistical analysis, categorical variables were assigned numerical values as follows: prosthetic outcome (success = 1, failure = 2); age (< 60 years = 1, ≥ 60 years = 2); gender (male = 1, female = 2); smoking status (current smoker = 1, former smoker = 2, never smoked = 3); implant length (short implant < 10 mm = 1, long implant ≥ 10 mm = 2); and type of prosthesis (single crown = 1, splinted crowns = 2, FPD = 3).

Variables with *p* < 0.05 in univariate analysis were entered into a multivariate logistic regression model to identify independent risk factors for prosthetic failure (cumulative risk). Kaplan–Meier curves with log-rank tests were used for survival comparisons. Logistic regression assessed associations with overall failure, whereas Cox proportional hazards regression evaluated the effect of risk factors on the timing of failure (time-to-event analysis). The Cox model was also used to analyze time-dependent effects. A *p*-value < 0.05 was considered statistically significant. The Cochran-Armitage trend test assessed linear trends between ordinal variables (e.g. Terheyden grades) and prosthetic failure.

## Results

### Overall prosthetic outcomes

Implant-supported prostheses were successful in 97 cases (success rate: 64.7%). The remaining 53 cases (35.3%) were classified as failures. The reasons for failure included implant mobility (28 cases), persistent pain (16 cases), bone resorption exceeding half the implant length (6 cases), and implant loss (3 cases).

### Univariate analysis of factors influencing prosthetic outcomes in patients with alveolar bone atrophy

Univariate analysis identified several factors as being significantly associated with the prosthetic outcomes of implant-supported restorations in patients with alveolar bone atrophy. These included patient age (χ² = 12.515, *p* < 0.001), gender (χ² = 13.940, *p* < 0.001), smoking status (χ² = 13.572, *p* < 0.001), implant length (χ² = 12.040, *p* < 0.001), and type of prosthesis (χ² = 14.226, *p* < 0.001). The detailed results of this analysis are presented in Table 1. Notably, the failure rates for Terheyden grades I through IV were 25.0, 29.4, 34.1, and 51.3%, respectively, showing a progressive increase with higher grade (Cochran-Armitage trend test *p* = 0.017).

**Table 1:** Univariate analysis of factors influencing prosthetic outcomes in patients with alveolar bone atrophy (n [%]).

Influencing factor	Prosthetic success group (*n* = 97)	Prosthetic failure group (*n* = 53)	χ^2^	*P*
Age (years)				
< 60	40 (41.2)	7 (13.2)	12.515	< 0.001
≥ 60	57 (58.8)	46 (86.8)		
Gender				
Male	62 (63.9)	17 (32.1)	13.940	< 0.001
Female	35 (36.1)	36 (67.9)		
Smoking status				
Current smoker	28 (28.9)	10 (18.9)	6.398	0.041
Former smoker	57 (58.8)	28 (52.8)		
Never smoked	12 (12.4)	15 (28.3)		
Diabetes mellitus				
Yes	41 (42.3)	28 (52.8)	1.539	0.215
No	56 (57.7)	25 (47.2)		
Bone quality (Lekholm & Zarb)				
B	40 (41.2)	14 (26.4)	3.892	0.143
C	19 (19.6)	16 (30.2)		
D	38 (39.2)	23 (43.4)		
Bone quantity (Terheyden classification)				
Grade I	27 (27.8)	9 (17.0)	6.571	0.087
Grade II	24 (24.7)	10 (18.9)		
Grade III	27 (27.8)	14 (26.4)		
Grade IV	19 (19.6)	20 (37.7)		
Surgical approach				
GBR	25 (25.8)	18 (34.0)	2.034	0.362
Maxillary sinus floor elevation	23 (23.7)	8 (15.1)		
Block bone graft	49 (50.5)	27 (50.9)		
Implant insertion direction				
Vertical	43 (44.3)	32 (60.4)	3.530	0.060
Tilted	54 (55.7)	21 (39.6)		
Implant length				
Short implant (< 10 mm)	27 (27.8)	30 (56.6)	12.040	< 0.001
Long implant (≥ 10 mm)	70 (72.2)	23 (43.4)		
Implant diameter (mm)				
< 3.6	25 (25.8)	11 (20.8)	2.215	0.330
3.6~4.2	39 (40.2)	28 (52.8)		
> 4.2	33 (34.0)	14 (26.4)		
Intraoperative complications				
Yes	22 (22.7)	16 (30.2)	1.021	0.312
No	75 (77.3)	37 (69.8)		
Postoperative complications				
Yes	15 (15.5)	11 (20.8)	0.670	0.413
No	82 (84.5)	42 (79.2)		
Type of prosthesis				
Single crown	23 (23.7)	28 (52.8)	14.226	< 0.001
Splinted crowns	46 (47.4)	12 (22.6)		
FPD	28 (28.9)	13 (24.5)		
Cuspal inclination (°)				
25	27 (27.8)	18 (34.0)	1.430	0.489
35	22 (22.7)	8 (15.1)		
45	48 (49.5)	27 (50.9)		
Occlusal scheme				
Group function	47 (48.5)	29 (54.7)	0.538	0.463
Canine guidance	50 (51.5)	24 (45.3)		
Oral hygiene (MPI score)				
0	27 (27.8)	11 (20.8)	2.748	0.432
1	31 (32.0)	18 (34.0)		
2	29 (29.9)	14 (26.4)		
3	10 (10.3)	10 (18.9)		

Note: GBR: guided bone regeneration; FPD: fixed partial denture; MPI: modified plaque index. The failure rates by Terheyden grade were: grade I: 25.0% (9/36), grade II: 29.4% (10/34), grade III: 34.1% (14/41), and grade IV: 51.3% (20/39), showing a progressive increase with higher grade (Cochran-Armitage trend test *p* = 0.017).

### Multivariate analysis of factors influencing prosthetic outcomes in patients with alveolar bone atrophy

Variables demonstrating statistical significance (*p* < 0.05) in the univariate analysis, namely age, gender, smoking status, implant length, and type of prosthesis, were included in a multivariate logistic regression model. Due to complete separation caused by smoking status, this variable was excluded from the final model. As shown in Table 2, multivariate logistic regression confirmed that short implant length (< 10 mm) was the only independent risk factor for prosthetic failure (OR = 4.418, 95% CI: 1.643–12.455, *p* = 0.004). Age ≥ 60 years (OR = 2.560, 95% CI: 0.856–7.892, *p* = 0.094), female gender (OR = 1.127, 95% CI: 0.417–2.886, *p* = 0.807), and prosthesis type (*p* > 0.05 for both comparisons) did not reach statistical significance.

**Table 2:** Multivariate analysis of factors influencing prosthetic outcomes in patients with alveolar bone atrophy.

Independent variable	β	SE	Wald	OR	*P*	95% CI
Age ≥ 60 years	0.940	0.561	2.808	2.560	0.094	0.856–7.892
Female gender	0.120	0.471	0.065	1.127	0.807	0.417–2.886
Short implant (< 10 mm)	1.486	0.515	8.324	4.418	0.004	1.643–12.455
Single crown (vs. FPD)	–0.833	0.555	2.252	0.435	0.135	0.143–1.290
Splinted crowns (vs. FPD)	–0.416	0.635	0.429	0.660	0.512	0.191–2.357

SE: Standard error; OR: odds ratio; CI: confidence interval.

Sensitivity analysis including Terheyden grade as a continuous variable in the multivariate logistic regression model showed that Terheyden grade was significantly associated with prosthetic failure (OR = 7.318, 95% CI: 2.994–19.963, *p* < 0.001). However, due to the limited number of events (*n* = 57), which allows the inclusion of only 5–6 variables based on the 10 events per variable rule, Terheyden grade was not included in the primary model to avoid overfitting.

### Survival analysis of factors influencing prosthetic outcomes in patients with alveolar bone atrophy

Survival analysis was performed using Kaplan–Meier curves with log-rank tests and multivariable Cox proportional hazards regression.

**Figure 1:**
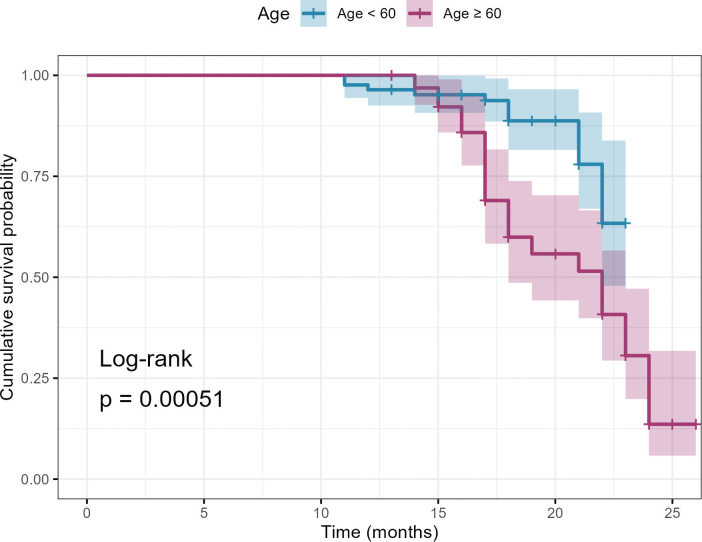
Kaplan–Meier survival curves comparing prosthetic failure-free survival between patients aged <60 years and ≥60 years with alveolar bone atrophy. Log-rank *p* = 0.010.

**Figure 2:**
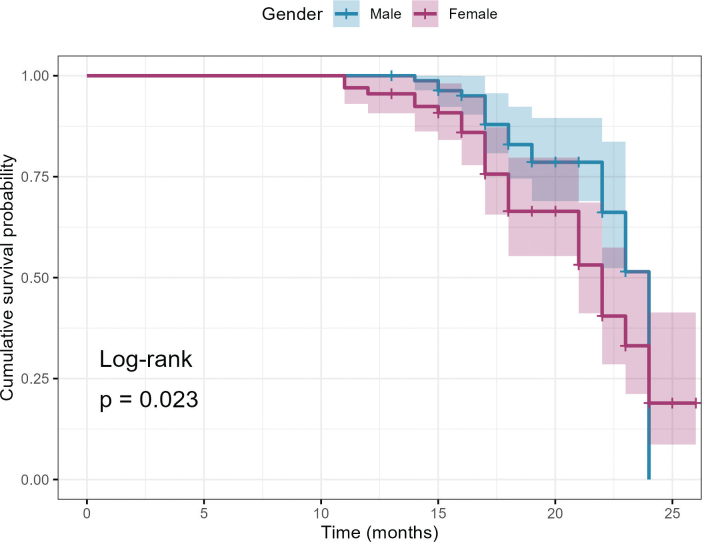
Kaplan–Meier survival curves comparing prosthetic failure-free survival between male and female patients with alveolar bone atrophy. Log-rank *p* = 0.023.

**Figure 3:**
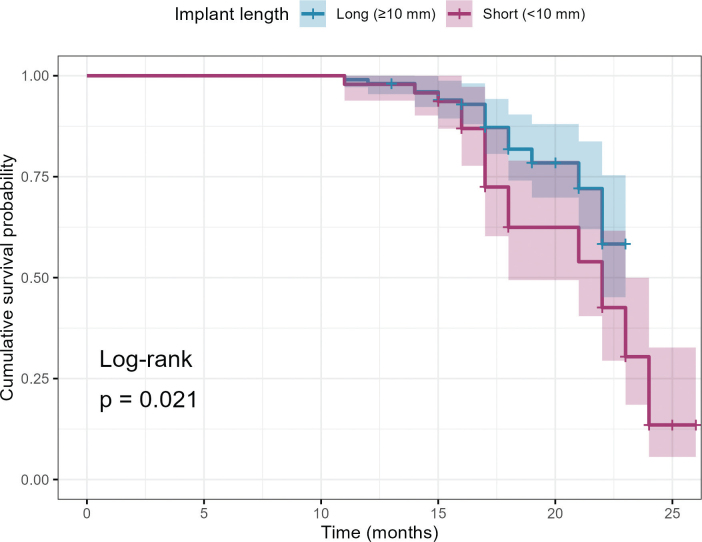
Kaplan–Meier survival curves comparing prosthetic failure-free survival between patients receiving short implants (<10 mm) and long implants (≥ 10 mm) with alveolar bone atrophy. Log-rank *p* = 0.021.

Kaplan–Meier analysis with log-rank tests (Figures 1–3): Patients aged ≥ 60 years had significantly lower prosthetic failure-free survival compared to those aged < 60 years (log-rank *p* = 0.010). Female patients showed a trend toward lower survival, but the difference did not reach statistical significance (log-rank *p* = 0.323). Patients receiving short implants (< 10 mm) also showed lower survival compared to those receiving long implants (≥ 10 mm) (log-rank *p* = 0.021).

Multivariable Cox regression (Table 3): After adjusting for age, gender, and implant length, only age ≥ 60 years remained an independent predictor of earlier prosthetic failure (HR = 2.558, 95% CI: 1.250–5.235, *p* = 0.010). Female gender (HR = 1.375, 95% CI: 0.731–2.586, *p* = 0.323) and short implants (HR = 0.959, 95% CI: 0.471–1.951, *p* = 0.907) were not significant independent predictors. The discrepancy between log-rank and multivariable Cox results for short implants suggests that the observed survival difference is largely explained by confounding factors, particularly age.

**Table 3:** Multivariable Cox proportional hazards regression analysis of factors associated with time to prosthetic failure.

Variable	HR	95% CI	*P*
Age ≥ 60 years	2.558	1.250–5.235	0.010
Female gender	1.375	0.731–2.586	0.323
Short implant (< 10 mm)	0.959	0.471–1.951	0.907

Note: HR: hazard ratio; CI: confidence interval. Multivariable model included age, gender, and implant length simultaneously.

## Discussion

This study, through a systematic analysis of follow-up data from 150 patients with alveolar bone atrophy, identified several key factors influencing the outcomes of implant-supported prostheses. Multivariate logistic regression identified short implant length as the sole independent risk factor for overall failure, while Cox regression revealed advanced age (≥ 60 years) as the only independent predictor of earlier failure. These findings update the existing understanding of short implants in the context of alveolar bone atrophy and provide new insights for clinical decision-making.

The 35.33% failure rate in this study is significantly higher than the 10–25% reported in similar studies on short implants and atrophic ridges [15, 16]. First, the study population had more severe bone atrophy, with 34.41% of patients being Cawood IV grade, which is higher than the 20–25% in other studies [4]. Severe bone atrophy reduces the bone quality and quantity, thereby impairing osseointegration. Second, the study period (2021–2022) was the initial stage of the department’s development of implant therapy for atrophic alveolar ridges. The failure rate in the first 6 months was 45%, while it decreased to 32% in the subsequent 18 months, indicating that the surgical proficiency of the operators improved over time. Third, although bone augmentation procedures were performed to achieve at least 1.5 mm of bone surrounding the implant shoulder in accordance with ITI recommendations, the severity of baseline alveolar atrophy (34.41% with Cawood grade IV) may have compromised the quality and extent of bone regeneration in some cases. Suboptimal bone regeneration, combined with the learning curve effect during the early study period, likely contributed to the higher failure rate observed in this cohort. The Cochran-Armitage trend test confirmed a significant linear trend between Terheyden grade and prosthetic failure (*p* = 0.017), indicating that bone quantity is a significant predictor.

The results identified an age of ≥ 60 years as an independent risk factor for implant failure. Elderly patients often exhibit physiological decline, including reduced bone metabolism, impaired microcirculation, and diminished cellular regenerative capacity. These age-related changes collectively compromise the process and quality of osseointegration. This observation aligns with the finding of Schwartz-Arad et al. [17], who revealed a positive correlation of increasing age with the risk of implant failure. However, other researchers, such as Abou-Ayash et al. [18], contend that advanced age itself is not an absolute contraindication for implant therapy, provided that indications are strictly followed and surgical protocols are optimized. The higher failure rate observed in our elderly cohort (73.68%) suggests that in the context of already compromised basal bone volume, the biological disadvantages associated with aging may be amplified. This is consistent with the recent meta-analysis by Schimmel et al. [19], which reported that the risk of implant failure in elderly patients with bone atrophy is 2.3 times higher than that in young patients. This underscores the need for more meticulous preoperative assessment and closer postoperative monitoring in this patient population. In our multivariable Cox regression analysis, age ≥ 60 years was the only significant predictor of earlier prosthetic failure (HR = 2.558, 95% CI: 1.250–5.235, *p* = 0.010). This indicates that while elderly patients did not have a significantly higher overall failure rate in logistic regression (*p* = 0.094), when failure did occur, it tended to happen earlier in older patients according to Cox regression (HR = 2.558, *p* = 0.010).

Our univariate analysis showed a significant association between smoking status and implant outcomes in patients with alveolar bone atrophy, with current smokers having a notably higher failure rate (56.14%) than former and never smokers. However, smoking did not qualify as an independent risk factor in multivariate logistic regression, likely due to masking by confounding factors (e.g. age-related declines in bone metabolism/healing, oral hygiene) that exert more direct effects. Consistent with Çankaya et al. [20], this high failure rate highlights the clinical importance of smoking intervention, as nicotine-induced vasoconstriction and carbon monoxide-reduced blood oxygen-carrying capacity impair implant-site blood supply, cause hypoxia, and inhibit osseointegration [19]. Former smokers exhibited intermediate risk, indicating smoking cessation improves outcomes – supported by a recent Quintessence International study showing ≥ 3 months of preoperative cessation reduces failure by 40%. The higher failure rate in our current smokers versus 15–25% in similar studies [4, 16] may reflect the synergistic detrimental effect of smoking and alveolar bone atrophy on osseointegration. Preoperative smoking cessation counseling/intervention should thus be integrated into treatment plans.

In univariate analysis, female gender was identified as a significant risk factor for prosthetic failure (*p* < 0.001). Two potential mechanisms may account for this gender-related disparity. First, perimenopausal and postmenopausal women experience a sharp decline in estrogen levels, which accelerates bone resorption, reduces bone mineral density, and impairs bone healing capacity [21]. Second, females generally have smaller jawbone dimensions, including narrower alveolar ridges and thinner cortical bone. When combined with alveolar bone atrophy, these traits further compromise bone volume and quality available for implant placement, resulting in insufficient bone–implant contact and increased stress concentration at the implant-bone interface [22]. However, after adjusting for age and other confounding factors in multivariable logistic regression (OR = 1.127, 95% CI: 0.417–2.886, *p* = 0.807) and Cox regression (HR = 1.375, 95% CI: 0.731–2.586, *p* = 0.323), female gender was no longer significant. This suggests that the apparent increased risk in female patients is largely explained by confounding with age, as female patients in our cohort were more likely to be in the older age group. Therefore, female gender alone should not be considered a contraindication for implant therapy. Clinicians should focus on age and bone quality rather than gender when formulating treatment plans for patients with alveolar bone atrophy.

Our findings indicated a significant association between the use of short implants and an increased risk of prosthetic failure. While short implants can obviate the need for complex bone augmentation procedures in cases of limited bone height, their relatively smaller bone-to-implant contact area may lead to concentrated stress distribution, particularly in low-density bone. This can exacerbate marginal bone resorption and elevate the risk of early failure. This observation aligns with the report by Benzaquen et al. [15], who noted that under poor bone quality conditions, the long-term survival rate of short implants was somewhat lower than that of standard-length implants. Nonetheless, some researchers, such as Tang et al. [16], argue that short implants can still achieve predictable outcomes when cases are carefully selected, and techniques like osteotome-mediated sinus floor elevation are employed. In our study, the failure rate of short implants in patients with Cawood IV grade bone atrophy was as high as 68.75%, which is much higher than the 30–40% reported in previous studies. In multivariable logistic regression, short implant length (< 10 mm) was identified as an independent risk factor for prosthetic failure (OR = 4.418, 95% CI: 1.643–12.455, *p* = 0.004). However, multivariable Cox regression showed that short implants did not significantly accelerate the time to failure (HR = 0.959, 95% CI: 0.471–1.951, *p* = 0.907). This suggests that while short implants increase the overall cumulative risk of failure, they do not shorten the time to failure compared with standard-length implants. Therefore, in patients with severe alveolar bone atrophy, short implants should be used cautiously, and bone augmentation should be prioritized whenever feasible. However, when bone augmentation is not possible due to patient factors or anatomical limitations, short implants remain a viable option with acceptable outcomes, provided that patients are appropriately counseled about the increased risk.

The survival curves from this study demonstrated that advanced age (≥ 60 years), female gender, smoking, and the use of short implants (< 10 mm) not only increased the short-term risk of failure but also exerted a progressively negative impact over longer follow-up durations. The significant rise in failure risk among elderly patients after 18 months may be related to the more pronounced effects of age-related declines in bone metabolism and healing capacity under medium- to long-term functional loading. The elevated failure rate observed in smokers as early as 12 months post-surgery further corroborates the inhibitory effect of tobacco on early osseointegration. In implant dentistry, failures are typically categorized as early failures (occurring before prosthetic loading or within the first year) and late failures (occurring after 1 year of function). In our study, most failures occurred as late failures. The sharp decline in survival curves after 20 months observed in female patients and short implant recipients is noteworthy. This delayed failure pattern may be explained by the following mechanisms: for female patients, the progressive decline in estrogen levels and its cumulative effect on bone resorption may take 18–24 months to significantly compromise implant stability; for short implants, the smaller bone–implant contact area may tolerate initial loading but gradually accumulate micro-damage and peri-implant bone loss over time, leading to late failure. This pattern differs from typical early failures (e.g. failed osseointegration), suggesting that risk factors for late failures may be distinct from those for early failures.

To further explore the discrepancies between univariate and multivariable analyses, we examined the confounding effect of age. Interestingly, while log-rank tests demonstrated significant survival differences for both female gender (*p* = 0.023) and short implant length (*p* = 0.021), multivariable Cox regression adjusting for age rendered both effects non-significant (*p* = 0.323 and *p* = 0.907, respectively). This indicates that the apparent associations between female gender and prosthetic failure, as well as between short implants and failure, are largely attributable to confounding by age. In our cohort, female patients and those receiving short implants tended to be older, and age emerged as the dominant predictor of failure. This finding has important clinical implications: when assessing risk in patients with alveolar bone atrophy, age should be considered the primary factor, and the apparent risks associated with gender or implant length may be overestimated without proper adjustment for age.

The findings of this study have several clinical implications. The proposed ‘triple risk overlap’ early warning model can help clinicians identify high-risk patients preoperatively. The identified key time points can guide targeted follow-up schedules. The study also emphasizes the importance of standardized bone atrophy classification and comprehensive data collection.

This study has several limitations. First, it is a single-center retrospective study with inherent selection bias. Second, some variables (e.g. surgeon experience, arch location, residual bone height, implant surface) were not collected, which may have introduced unmeasured confounding. Third, clinical parameters such as probing depth and keratinized mucosal width were not systematically recorded. Fourth, implant length was recorded only as short (< 10 mm) or long (≥ 10 mm), preventing re-stratification into three categories. Fifth, the composite outcome was treated as binary without weighting individual domains. Sixth, while log-rank tests showed significant survival differences for gender and implant length, multivariable Cox regression revealed these effects were confounded by age.

Future prospective multi-center studies should incorporate more comprehensive variables and longitudinal radiographic assessment to further clarify the mechanisms underlying implant success or failure in patients with alveolar bone atrophy.

Seventh, although sensitivity analysis confirmed a significant association between Terheyden grade and prosthetic failure (OR = 7.318, *p* < 0.001), this variable was not included in the primary multivariable model due to sample size limitations.

## Conclusions

In patients with severe alveolar bone atrophy, bone augmentation should be prioritized over short implants. Advanced age (≥ 60 years) is the dominant independent predictor of prosthetic failure, with key time points at 12, 18, 20, and 24 months postoperatively. The apparent associations of female gender and short implant length with failure are largely confounded by age. Clinical risk stratification should prioritize patient age over implant length or gender. Logistic and Cox regression provided complementary insights: implant length affects cumulative failure risk, whereas age influences the timing of failure. These findings require validation in large-scale, multi-center prospective studies.

## Data Availability

The data presented in this study are available on request from the corresponding author.
